# Supplementary material for: Bioinformatics and Expression Analysis of the Chitinase Genes in Strawberry (Fragaria vesca) and Functional Study of FvChi-14
Source: Plants (Basel). 2023 Apr 3;12(7):1543. doi: 10.3390/plants12071543 (PMC10097121; doi:10.3390/plants12071543)
Supplement: Supplementary file 1 [file plants-12-01543-s001.zip › plants-2289709-supplementary.pdf]

```
1   ATGCAGGCCAAGTGGATTCTTCTGCTTTTCTCAATCATCCTCGTA
     M  Q  A  K  W  I  L  L  L  F  S  I  I  L  V
46  GTGCAAAATGTGAACGGTGAAGATGAGTCGTTATCATCGTCTGTG
     V  Q  N  V  N  G  E  D  E  S  L  S  S  S  V
91  AAGCCATTGGTGAAGATTGTAAGGGGCAAGAAGCTATGTGACAAG
     K  P  L  V  K  I  V  R  G  K  K  L  C  D  K
136 GGTTGGGAGTGCAAGGGATGGTCTGCTTATTGTTGTAACCATACT
     G  W  E  C  K  G  W  S  A  Y  C  C  N  H  T
181 ATTTCAGACTACTTTCAGTCTTACCAATTTGAGGACCTTTTCTCT
     I  S  D  Y  F  Q  S  Y  Q  F  E  D  L  F  S
226 AAGCGCAACTCGCCTGTGGCGCATGCAGTCGATTTCTGGGACTAC
     K  R  N  S  P  V  A  H  A  V  D  F  W  D  Y
271 CATTCTTTCATTACTGCTGCGGCTGGGTACCAGCCTCATGGCTTT
     H  S  F  I  T  A  A  A  G  Y  Q  P  H  G  F
316 GGTACCACTGGAGGCAAGCTCCAGGGGATGAAAGAAGTTGCAGCT
     G  T  T  G  G  K  L  Q  G  M  K  E  V  A  A
361 TTCCTTGGTCATGTTGGCAGCAAAACTTCATGTGGATATGGGGTA
     F  L  G  H  V  G  S  K  T  S  C  G  Y  G  V
406 GCTACAGGAGGACCATTGGCTTGGGGTCTTTGCTACAACAAGGAG
     A  T  G  G  P  L  A  W  G  L  C  Y  N  K  E
451 ATGAGTCCTAACCAGTTGTATTGTGACGATTATTTCAAATACACG
     M  S  P  N  Q  L  Y  C  D  D  Y  F  K  Y  T
496 TATCCTTGTGCTCCTGGAGCTTCATACCACGGCCGTGGTGCCTTG
     Y  P  C  A  P  G  A  S  Y  H  G  R  G  A  L
541 CCACTATACTGGAACTACAACTATGGAGAAACAGGAGAAGCCCTG
     P  L  Y  W  N  Y  N  Y  G  E  T  G  E  A  L
586 AAGGTGGACTTGTTGAACCATCCAGAATACATAGAACAGAATGCT
     K  V  D  L  L  N  H  P  E  Y  I  E  Q  N  A
631 ACTCTGGCCTTTCAGGCTGCAATTTGGAGGTGGATGACTCCAGTG
     T  L  A  F  Q  A  A  I  W  R  W  M  T  P  V
676 AAGAAGAACGTTCCTTCAGCCCATGATGTCTTTGTTGGGAAATGG
     K  K  N  V  P  S  A  H  D  V  F  V  G  K  W
721 AAACCTACCAAGAATGACACATTGGCCAAAAGGGTTCCTGGATTT
     K  P  T  K  N  D  T  L  A  K  R  V  P  G  F
766 GGCACAACCATGAATGTTCTCTATGGGGAGCAAACTTGTGGTCAA
     G  T  T  M  N  V  L  Y  G  E  Q  T  C  G  Q
811 GGTGATGTTGACTCCATGAACAACATTGTCTCGCATTACCTGTAT
     G  D  V  D  S  M  N  N  I  V  S  H  Y  L  Y
856 TACCTTGATCTTATTGGTGTTGGCCGCGAAGAGGCAGGTCCTCAT
     Y  L  D  L  I  G  V  G  R  E  E  A  G  P  H
901 GATGTGCTCAACTGCGCTGAGCAAGAAGCTTTCGAGCGTTCCTCT
     D  V  L  N  C  A  E  Q  E  A  F  E  R  S  S
946 TCTTCTTCATCATCTTCATCTTCTTGA
     S  S  S  S  S  S  S  S  *
```

Figure S1. The deduced amino acid and nucleotide sequence of *FvChi-14*. The underlined part denotes chitinase domain.

Table S1. Details on the primer sequences used.

Gene name	Forward primer sequence	Reverse primer sequence
FvChi-1	CTCCACCCAACAGACATTC	GAGAAGACTGGTTCAGCATTG
FvChi-2	CCTCTTCTCCCTCACTACCATC	ATGCCTTGTCGTCCTTGTG
FvChi-3	TTGGGATTGTAGGCAAC	TAGTCTTTTCACTGCTATTTGCTG
FvChi-4	TGATTTCCCACCTTCAGC	GAGCAGAGTCGTTTCATTTG
FvChi-5	GAGAGTACTTAAGTCAATTGGCAAC	ACCCTCTCCAAGCTTATTCTTACT
FvChi-6	CACCGAGATGACCAACTTC	GCTGTAGTAATCCGCATTGC
FvChi-7	ACAATGCGGTAGACAAGCC	GGTTGGTGATGAGGGAGTAG
FvChi-8	GTCACAACCTTACTGTCCATCTG	GGCTTTCCTGACTTGCTTG
FvChi-9	CTCATCACCCGTTTGAAAG	CGGAGTCCTAACCTTGTCAG
FvChi-10	GTCAAACCTCGCTCTGTTG	GTCAAAGCACATCGGACTC
FvChi-11	TTGAATGTGGTAAGGGCTC	TAAGTAGCGAGTCCAGACCC
FvChi-12	CCAATAGATTCCCGAGTCG	GCTGAGATTCTTTGTGTCCC
FvChi-13	GGGAATACATAGGAGCCGAG	TCCCAGAACGCATTGAAG
FvChi-14	TGAAGGTGGACTTGTTGAAC	GTGTCATTCTTGGTAGGTTTCC
FvChi-15	GGACCGATTCAACTATCTTGG	TTGCTCCGTCATCCAGAAC
FvChi-16	TGTATTCATTCAAGCCCTCG	CGATGGCAAAGGACAGAAG
FvChi-17	TTGACATTGAGGGAGGGTC	GGCAAGGAGGGTTGTTGTAG
FvChi-18	CATTGACTACGACACCTCAGG	GGTTGAAGTAAGCAGAGCCAC
FvChi-19	GTGAACATAGCCTTCCTCAAC	AAAGAGTAGTTTCCGATGCC
FvChi-20	CCAAAGGCATCAAGGTCATAC	AGAACAGCATCTCCCAACG
FvChi-21	AAGCAATGGACTTCAGCC	CGGAAGGACTGTTGTAGTGAG
FvChi-22	TGCTGATGGGAACTATGGAC	AACTGGTCTGGAAATGGGC
FvChi-23	TGCCTCTTACACCGATTTC	GGTAGTCTGTTCTGCTTCCG
Fvactin	GCCAGAAAGATGCTTATGTCGGTG	TGGGGCAACACGAAGCTCAT

Gene name	Forward primer sequence	Reverse primer sequence
FvChi-14-clone	CGGGATCCCGATGCAGGCCAAGTGGATTCTTCTG	GGACTAGTCCTCAAGAAGATGAAGATGATGAAGA
FvChi-14-SL	CGGGATCCCGATGCAGGCCAAGTGGATTCTTCTG	TGCTCTAGAGCAAGAAGATGAAGATGATGAAGAAGA
FvChi-14-PE	CGGGATCCCGATGCAGGCCAAGTGGATTCTTCTG	TCCCCCGGGGGATCAAGAAGATGAAGATGATGAAGA

Table S2. Motif sequences identified found in *FvChi* genes.

Motif	Length (aa)	Sequence
1	41	SMLSQSSWRKNFIDSSIRIARLYEFDGJDJDYEHPRTVPNT
2	50	NDDNWVLSRAAAEEEGGRQNKKQLLVIVLVTISMFIILLGTIMCYMQRRV
3	46	KKVYLTAAPQCPFPDAYVGNALSTGLFDYVWVQFYNNPPCQYTSGB
4	41	WGQNGNEGTLSKTCASGNYEFVNIAFLSAFGNGQTPVINLA
5	50	HHMQVVDTETYPIDSIKKNLNWVHLLAYDYYLPKMENYTHPHAALYDPIS
6	41	MYNDTYVSNYCTIGSSWINFDDVEAIKAKVSYAKEMGLLGY
7	41	PEYPCAPGKSYYGRGPIQLSWNYNYGQAGKAJGFDGLNNPE
8	21	FTSVVKCKNSNVKVLLSJGGG
9	41	NGTNTNSRVKDLINRGLPASKLVLGLPYHGYGWTVLNPSNH
10	50	YCTKREEKMLIYEYMPNKSLDLYLFDPSRCDLLHWEQRVHIIEGVIQGLL
11	38	KAGYHYPGDEFPASTIDTTLFTHLLCAFAYINSSSYQL
12	50	FASIAPYDDDDVQPHYLALWKKYGQLIDYVNFQFYAYDKGTTVSQFIKYF

Motif	Length (aa)	Sequence
13	29	LGTFLKZWRSAIDAEASFSGKPALLLTAA
14	21	STSRPEGDAVLAGVDFDTEGG
15	21	LVAIDPSVEFKFILWFWKTYD
16	50	HDVIVGRWNPTEADIAAGRTPGFGTITNIINGGLECGIGSDARVNDRIGY
17	41	PENGFFTACARLKSZGKLHGIFIWSADDSKKAGFRYEKQSQ
18	50	MNPKISDFGLARAFRKNELEANTGRIVGTYGCVPPEYVRRGIYSMKFDVY
19	41	VLLLQIISGKRSSCFYGIDGNLNLLEYAYELWKEGQGMDFV
20	29	HRDDSNAPALQFYSYASFIAATNNFPAFG

Table S3. Signaling hormones and stresses-related *cis*-acting elements in the promoter regions of *FvChi* genes.

Gene name	TCA-element	CGTCA-motif	TGACG-motif	TCT-motif	ABRE	TGA-element	ARE	G-box	Box 4	MBS	TC-rich repeats	LTR	TATC-box	circadian
FvChi-1	3	2	2	1	2	1	2	3	0	0	1	0	0	0
FvChi-2	0	2	2	4	0	1	0	0	1	0	0	1	2	0
FvChi-3	1	0	3	2	0	2	0	1	0	1	1	2	0	1
FvChi-4	1	0	1	0	0	1	0	2	0	1	0	2	1	0
FvChi-5	1	0	3	0	0	2	0	1	2	1	1	2	0	1
FvChi-6	0	0	1	0	0	1	3	4	2	1	1	0	0	0
FvChi-7	0	0	1	0	0	0	4	10	1	0	1	0	1	0
FvChi-8	0	0	3	0	0	1	2	1	4	0	0	1	0	1

Gene name	TCA-element	CGTCA-motif	TGACG-motif	TCT-motif	ABRE	TGA-element	ARE	G-box	Box 4	MBS	TC-rich repeats	LTR	TATC-box	circadian
FvChi-9	1	0	0	0	0	1	4	1	1	1	0	1	0	0
FvChi-10	0	2	2	0	0	0	1		1	0	0	1	0	0
FvChi-11	0	2	2	1	2	0	3	3	1	1	0	0	1	1
FvChi-12	0	0	2	2	1	1	5	2	1	1	2	0	0	0
FvChi-13	0	1	1	0	3	1	3	4	4	3	2	1	0	0
FvChi-14	1	2	2	1	7	0	2	8	2	0	0	0	0	0
FvChi-15	2	1	1	1	1	0	3	3	0	1	1	0	0	0
FvChi-16	0	0	0	1	0	0	0	0	0	0	0	3	0	0
FvChi-17	0	0	0	0	1	0	2	1	2	3	1	0	0	1
FvChi-18	0	0	0	2	2	0	1	4	2	0	0	0	1	0
FvChi-19	0	0	0	0	2	0	1	2	3	1	0	0	0	1
FvChi-20	0	1	1	1	1	0	3	1	0	11	1	1	0	0
FvChi-21	0	2	2	3	9	2	1	11	2	1	1	1	0	0
FvChi-22	1	2	2	2	2	0	5	1	2	0	1	1	0	0
FvChi-23	2	2	2	0	0	0	2	4	3	0	0	0	0	0

Table S4. Functions of the *cis*-elements found in the promoters of *FvChi* genes.

Motif	Function
TCA-element	cis-acting element involved in salicylic acid responsiveness
TGACG-motif<br>CGTCA-motif	cis-acting regulatory element involved in the MeJA-responsiveness
TCT-motif	part of a light responsive element
ABRE	cis-acting element involved in the abscisic acid responsiveness

Motif	Function
TGA-element	auxin-responsive element
ARE	cis-acting regulatory element essential for the anaerobic induction
G-box	cis-acting regulatory element involved in light responsiveness
Box 4	part of a conserved DNA module involved in light responsiveness
TC-rich repeats	cis-acting element involved in defense and stress responsiveness
LTR	cis-acting element involved in low-temperature responsiveness
TATC-box	cis-acting element involved in gibberellin-responsiveness
